# Book Reviews

**Published:** 1822-09

**Authors:** 


### CRITICAL ANALYSIS
### OF
### ENGLISH AND FOREIGN LITERATURE
### RELATIVE TO THE VARIOUS BRANCHES OF
### Medical Science.

Quæ laudanda forent, et quæ culpanda, vicissim

Illa, prius, cretâ; mox hæc, carbone, *notamus.*—PERSIUS.

### DIVISION I.

### ENGLISH.

ART. I.—*A History of a severe Case of Neuralgia, commonly called Tic Douloureux, &c.; with Observations on that Complaint.* By G. D. YEATS, M.D. F.R.S. Fellow of the Royal College of Physicians, &c. &c. &c. Burgess and Hill, 1822.

ART. II.—*Cases of Neuralgia Spasmodica, commonly called Tic Douloureux, successfully treated.* By BENJAMIN HUTCHINSON, Fellow of the Royal College of Surgeons of London, &c. Second Edition. Longman and Co. 1822.

ART. III.—*Observations on the Nervous System.* By JOSEPH SWAN, Member of the Royal College of Surgeons, and Surgeon to the Lincoln County Hospital. Longman and Co. 1822.

BEFORE we present our readers with an analysis of the works placed at the head of this article, it may not be unprofitable to take a rapid view of some of the principal facts connected with the subject of which they treat. Tic douloureux is not dangerous to life; yet we believe there are few, who have ever experienced its tortures, who would not willingly exchange it for some more perilous, but less painful, ailment. The sympathy excited towards its victims has given an additional stimulus to professional industry, and much attention has of late years been devoted to its investigation: many experiments have been tried, and many learned dissertations have been written, though without much effect, either in improving the practice or elucidating the pathology. Important facts, however, have been collected regarding the functions of the nerves; and, besides, in searching for an unknown remedy, it is of no small importance to be aware of what has been found unavailing, as it naturally directs our inquiries into new, and therefore more likely, channels. On this account, we willingly embrace the opportunity of calling to the memory of our readers some of the results which have been deduced from these investigations.

The history of tic douloureux may be regarded as of modern date: we find, indeed, that an essay was published, so early as 1672, by DANIEL LUDOVICO, "De Dolore superciliari acerbissimo periodico;" but whether this may have referred to periodical head-ach, or to that affection of the superciliary nerve since known by the name of tic douloureux, we are unable to say. However this may be, no doubt can exist of the account given by the celebrated Dr. JOHN FOTHERGILL, "of a painful Affection of the Face," in 1776, being the first which appeared in this country; and, indeed, the symptoms are described with a force which (resulting from actual observation,) has scarcely been surpassed by his successors: nor do we regard his pathology, which attributed the disease to acrid matter irritating the nerves, as at all inferior to the speculations of the present day upon this subject. The attention of medical men having once been excited, and the disease being no longer confounded with tooth-ach and rheumatism, a variety of essays were soon published regarding it, of which it would be foreign to our purpose to speak individually. The term, tic douloureux, seems at first to have been confined to the affection of the nerves of the face; but, as other nerves were found to be subject to a disease exactly similar, the same appellation was extended to them. The nerves which practical writers have enumerated as most liable to this disease, either in their principal trunks or minute branches, are the superciliary, infraorbitary, the submaxillary, the portia dura of the seventh pair, the cubital, the intercostal, the lumbar, the anterior crural, the spermatic, and the sciatic. Of these, the most frequently affected with true tic douloureux are the three first; and next in frequency come the nerves of the thigh: some, on the contrary, are very rarely affected, such as the intercostal; one instance of which is quoted by CHAUSSIER, on the authority of SIEBOLD, and another is to be found in the second edition of Mr.

Hutchinson's work. The only example with which we are acquainted of the spermatic nerve being supposed to be the seat of the disease, is mentioned on the authority of M. Barras, and we confess it appears to us very doubtful: the pain was attended with considerable inflammation of the testicle, and was cured by the application of moxa along the course of the spermatic cord.

In the above list we have enumerated the nerves as we find them mentioned by different authors, without pledging ourselves for their accuracy: one nerve obviously presents matter for further investigation, and involves much physiological interest,—we mean the portia dura. If it should be proved that practical writers (among whom is Mr. Hutchinson,) are right in attributing the seat of disease in some instances to this nerve, it would be an awkward dilemma for those who confine its functions to the office of coadjutor in the process of respiration. We have heard a case in which Sir Astley Cooper lately divided the portia dura, spoken of as one of tic douloureux; but personal acquaintance with the patient enables us to contradict the statement. The lady in question had a tumor in the site of the parotid gland, which was removed, six or seven years ago, by Dr. Thomson, of Edinburgh; at which time some branches of the portia dura were divided, and a considerable degree of paralysis of the side of the face was the consequence. This loss of power does not seem to have been restored to the same extent as when the twigs of other nerves are divided, as the writer remembers the patient in question to have remained, during the period above mentioned, with but little improvement. The tumor having again attained an inconvenient size, was recently extirpated by Sir A. Cooper, and a more decided palsy of the face has resulted.

To the examples above mentioned, some authors are disposed to add all those painful affections of nerves arising from external injury, (as in bleeding,) or internal changes, (as the formation of tumors, either in the nerves themselves, or in their vicinity, so as to irritate them by pressure;) but at this rate there seems no limit to the term, and, as all sensation necessarily requires the medium of nerves for its communication, it would be as well to denominate every pain at once, tic douloureux. The nerves enumerated are principally those which are situated near the surface, with comparatively little covering either of fat or common integument: this, however, does not obtain universally,—the posterior crural forming a remarkable exception; nor, indeed, is it easy to conceive why any nerve capable of communicating pain may not, under particular circumstances, become affected with this disease. Accordingly, examples are to be found of painful affections of internal organs, supposed to resemble the neuralgia, or tic douloureux, of external nerves; and Delpech mentions an instance of the disease affecting the uterus and rectum. Although we have no doubt of the accuracy of M. Delpech, the disease having likewise manifested itself in the external distribution of the iliac nerve, yet such explanation of pains confined to internal parts ought to be received with much caution, particularly as the adoption of the tonic plan of treatment, recommended by Mr. Hutchinson, seems likely, and with justice, to gain the preference over others.

An attack of tic douloureux is sometimes, though not generally, preceded by some symptom, such as itching about the course of the nerve, which gives warning of its approach. Sometimes the pain comes on gradually, (as in an instance now under our observation,) adding to its tortures the horror of anticipation; at others, its attack is as sudden as it is violent. When it is once established, the peculiar intensity of the pain, in the site of a nerve, for the most part, points out unequivocally the nature of the complaint; some idea of the pain attending which may be formed from the forcible description of one of Mr. Hutchinson's correspondents. " It is not, I should conceive, possible," says he, " for any one who has not had some personal experience of this malady to form the least idea of the different effects it produces, some of which I will endeavour to enumerate. It sometimes commences with a slight corruscation, or ticking, somewhat similar to that of a pendulum, whence it may probably derive its name. It is afterwards succeeded by a shock more violent than that of an electrical machine, but of much longer duration. A red-hot salamander laid upon the head may afford some resemblance of the effect it sometimes produces. At other times it may convey some idea of the operation of an incision knife or tomahawk, the lancets of a cupping instrument being nothing compared to it. Sometimes you may imagine minute-guns passing through the head for a considerable length of time. The patient may, at others, suppose his head to be laid open with a battle-axe, and the brain exposed to a dreadful north-eastern blast."

When we turn from a description such as this, and search our books on pathology for the cause of so much suffering, we find them lamentably deficient: not only are we ignorant of those peculiar morbid changes which produce the disease, but even the parts changed, or supposed to be so, are disputed; some referring the seat of disease to the nerve, others to the brain. So far as we have considered, and can judge on this subject, we are inclined to believe the seat of disease generally exists in the nerve itself. The obvious proof is—divide the nerve, cut off the communication with the brain, and the pain ceases. This, at first sight, appears unanswerable; but then it may be asked, if we cut the infraorbitary nerve, and the pain ceases, how can it be shown but that it does so because the communication is cut off between the affected part and a morbid action in the brain, which requires the extremities of that nerve for its development in the sensation of pain; and, besides, division of the nerves, so far from relieving in some cases, aggravates the pain. With regard to the latter objection, we conceive the mischief to proceed from the division being made at a point which does not intercept the communication between the seat of disease and the brain; as appears to have been the case in the operation for injury of the median nerve, the history of which is detailed by Sir E. Home, in the Philosophical Transactions for 1801. With respect to the former, it would seem that, when irritation exists at a point nearer the brain than that to which the sensation of pain is referred, removal of the seat of pain altogether does not necessarily alter the sensation. A gentlemen of much celebrity in our profession cut his finger last winter; the point of it remains insensible to the touch of external bodies, and yet is liable to acutely painful sensations; showing that pain is referred to a part, the nerve of which giving the sense of touch is either not continuous, or altered in structure, by the process of re-union. But a more familiar and striking example is, that a man, sitting by his fire-side in London, shall have pain in the great toe of his foot, the leg having been left at Waterloo seven years ago. As this demonstrates that the brain is capable of referring pain to a part which does not even exist, so, if the seat of tic douloureux was in the brain, we should not expect such sudden and complete cessation of pain to follow the division. It is true such relief does not invariably follow; but this we believe to arise from an error in the part to which the disease is referred. A tumor in the groin will sometimes cause pain in the foot; but, to remove this, it would be absurd to divide the nerve between them. Another singular fact is, that the pain seems occasionally referred to a part of the nerve nearer the sensation than the seat of the disease. In the case of Richard Brag, related by Mr. Pring, in his excellent "View of the Relations of the Nervous System in Health and Disease," the second branch of the fifth pair was affected with tic douloureux; the pain was most acute in the portion of the nerve within the orbit, and extending towards the lower jaw,—that is, nearer the brain than the infraorbitary foramen,—yet its division at this point completely removed the symptoms. "This fact," says Mr. Pring, "furnishes a good proof of the locality of the disease, and is an example of the assumption that the origin of tic douloureux *is in the nerve, and not in the brain.*"

If, however, we grant that the disease is in the nerve, we cannot prove it, by demonstrating any change in its structure. Numerous examinations have been made. It is absurd to object that the patients seldom die, and therefore that opportunities are few; the examinations have been made under much more favourable circumstances, for the nerve has been laid bare and divided,—nay, portions of it cut out, while the patients were alive, and labouring under paroxysms of the disease; yet no morbid changes have been found, or none so uniform as to give any hint by which we may detect the *ratio symptomatum.* Chaussier and Bichat mention having occasionally found the femoral nerve with some degree of varix and œdema; but whether this change was cause or effect, they pretend not to determine. Inflammation is so frequent a cause of pain, that it naturally suggested itself, and, the neurilema having been fixed upon for its seat, the explanation was deemed at once obvious and satisfactory. Unfortunately, however, more extended observation did not confirm this opinion. We say unfortunately, because inflammation is an action which we are accustomed to contemplate,—which we flatter ourselves we understand,—and the effects of which we are unquestionably, in a great measure, acquainted with, and enabled to controul. Yet it is certainly a very limited view which would lead us to regard pain in a nerve as necessarily arising from inflammation; for some of the most acute pains of which we are susceptible arise from other causes: the pain, for instance, of a blow, or, to take a less exceptionable example, those stiches we frequently experience in different parts of the body, which come on and go off with a rapidity which precludes the possibility of their originating in any permanent cause. Indeed, the suddenness of the invasion, and ceasing of an attack, of tic douloureux, are of themselves sufficient grounds for the rejecting this explanation of its pathology. Pugal talks of erethism of the nerves; Fothergill of a gouty humour; and the followers of Broussais discover the acrimony of cancer either in the nerve or its sheath. Of all these hypotheses we entertain the same opinion,—they are *vox et preterea nihil;* and yet there must be something alluring in this sort of speculative inquiry, for two of the writers before us have indulged in it. Mr. Swan attempts to account for the attacks of tic douloureux being periodical, in this manner: "It may be," says he, "that a nerve cannot, at first, bear a diseased action continually without rest, any more than it can a healthy one; and therefore the diseased action, after a certain period, ceases to make any impression, or at least a much fainter one. But, after this rest, the nerve again acquires power, and is again fitted for the same action." Now, in answer to this, we would remark that, if nerves are capable of giving a continued sensation of pain in other structures, (as they unquestionably are,) it is impossible to suppose that a diseased action, in themselves, will cease to produce an impression merely from fatigue, particularly as the duration of the pain is often very short; and in other instances, as inflammation of a nerve from external injury, the pain does not intermit, but is continued. Again, he cannot help supposing "that in tic douloureux there is a sudden irregular action of the blood-vessels of the nerve," which causes the violent pain, in the same way as dizziness is produced in the eye. Now, as we cannot see the minute vessels of the nerve during this supposed action, nothing can be more difficult than to bring any direct proof against it,—except to bring any in its favour. If, however, we have recourse to analogy, we shall find it does not support Mr. Swan's doctrine. We do not deny that increased local actions of blood-vessels may suddenly occur; but we believe such actions do not long remain entirely local, and therefore that we may judge of the state of vessels beyond our means of direct examination by those within our reach. Thus, we cannot see the minute vessels in a whitlow; but, when we find the artery of the finger pulsating several times in the minute more than the same vessel at the wrist, we have legitimate grounds for believing the vessels in the whitlow to be in a corresponding state of excitement. But this state does not continue without affecting the artery at the wrist, likewise, to a certain extent, or even the general system; whereas, in tic douloureux, there is often, even when the nerve is superficial, no degree of increased action apparent. Neither do we admit the validity of the argument founded on the fact that Mr. Hunter had to apply a ligature to the musculo-cutaneous nerve, to stop a hemorrhage, and that he (Mr. S.) had seen the external popliteal nerve bleed profusely. These cases prove that there are painful affections of nerves, in which the blood-vessels become enlarged: with regard to tic douloureux, they prove nothing.

The opinions of Dr. YEATS seem directly opposed to Mr. Swan's: he informs us that, in the case which he has related at full length, the veins of the affected leg were empty, and asks, "Does this condition of the veins, in a part under such excruciating pain, give any idea of the particular state of its nerves?" and again, "It would seem that the arteries did not sufficiently propel their contents to the veins." Thus we have two speculations with regard to the condition of the blood-vessels in tic douloureux,—one, that their action is increased, the other, that it is diminished; and we leave our readers to choose between them. We do not presume to deny that some change of structure may take place in nerves affected with tic douloureux; we only assert that the nature of such change has never been demonstrated, and that it is not necessary that it should exist at all, because pain occurs in other parts, under circumstances which preclude the possibility of its being attended with change of structure. There are many cases where departure from natural organization has been observed in nerves, and a familiar instance of this is presented in their simple division; it resting upon the authority of MEYER, PRING, and others, that a change of structure occurs at the place of reunion. But, where this is observed, it is accompanied with a derangement of function which is continued. We mean, that although the part, the functions of which have become impaired, may eventually recover its energy, yet that the affection is not intermittent, as in tic douloureux.

As it appears, then, that we are yet in the dark respecting the proximate cause of the disease, we cannot, in practice, avail ourselves of any indication of cure, which the possession of this secret might be expected to afford. Accordingly, the treatment has been purely empirical; and, on looking over the list of remedies which have found their supporters, and whose claims are, of course, backed by *well-attested cures*, we find the simplest manner of describing them to be a reference to the whole range of the materia medica. No medicine has been deemed too deleterious, none too insignificant,—no operation has been deemed too dreadful, none too gentle;—all has been tried, from arsenic to rhubarb,—from scarification with red-hot knives, to the more harmless application of the metallic tractors. Among these, the most obvious analogy points to narcotics,—the means which lull pain in other instances. These have been used in every dose which prudence would allow; and opium, hyoscyamus, conium, and, more lately, belladonna and the sulphate of quinine, have been recommended. Of these, the only one, the exhibition of which in tic douloureux has fallen under our own immediate observation, is opium; and the inference we have drawn from our limited experience is, that it does not afford relief during the acute paroxysm of an attack, unless administered in such large and repeated doses as to bring on, and keep up, a state bordering upon stupor. Of all the remedies, however, carbonate of iron at present stands most conspicuous, from its novelty, and from the testimonies, both numerous and respectable, given in its favour. The evidence is before the public, and they must judge for themselves: our province is to give some of the suggestions that strike us on the perusal of different works; further than this we have neither the presumption nor the wish to influence the opinion of our readers. We think it but fair, however, to add one to the list of cures: a case having recently fallen under our immediate observation, which was distinctly marked as an affection of the nerves, both by the acuteness of the pain and its situation, affording an example, which seems not to be common, of the disease attacking both sides of the face, and the exit of four branches at once. The benefit from the use of the iron was decided; its permanence remains to be proved.*

The general failure in the operation of internal remedies to remove the disease, naturally led to the idea of destroying the seat of pain, or interrupting its communication with the brain; and the part which one of our authors (Mr. Swan,) has taken in investigating the phenomena attending the division of nerves, renders it but justice to him to take some notice of this very interesting subject. The idea of dividing the nerve supplying a part affected with pain is not of modern date, although we believe Marechal to have been the first who practised this operation for the relief of tic douloureux; since then it has been done so frequently as to render any enumeration of examples altogether unnecessary. The relief given by this operation was so complete, as to lead to the most sanguine hopes of its permanent effect. But it was soon found that the disease, although relieved for a time, returned again in proportion as the re-union of the nerve enabled it to discharge its usual functions. In order to remedy this evil, it was advised to cut out a portion of nerve; by which means it was supposed its continuity must be entirely and permanently destroyed. An interesting case of this kind is given by Mr. Abernethy, in which a portion of the nerve of the finger was removed, yet the sensibility of the part became restored; and Mr. Pring has mentioned a case in which *three inches* of the median nerve was removed: the wound healed by the first intention, and the patient was discharged at the end of two months. "During the last three weeks of his abode in the hospital, the condition of the arm had undergone a visible improvement: its motions were in a great measure restored, and the sensibility of it was likewise considerably augmented." It is added that, in six months after the operation, the powers of the arm were so far restored "that the man sustained little or no inconvenience in the use of it."

The restoration of sensibility after the division of a nerve has been, in general, supposed to arise from its re-union, and various means have been adopted to prevent this effect. In the case of Marechal, to which we have alluded, the pain returned at the end of two years; and M. Andrè applied caustic to the site of the nerve, by which means the disease was cured. This operation, in its various forms of caustic, cautery, and moxa, has since been frequently employed in France; and we are inclined to believe, could we divest ourselves or our patients of the horror it naturally excites, would be found more effectual

* See the Report of prevailing Diseases, inserted in this Number.

in destroying the nerve than either simple division or excision. The insufficiency of the former is, indeed, universally admitted; and, with respect to the latter, it has been found that the distance to which nerves will send new branches, to restore their continuity with the divided portion and contiguous parts, is such as could not have been at all anticipated. Yet there is a difficulty still more formidable than this to overcome; for, in the case cited above from Mr. Pring, it is impossible to believe that, after the removal of so considerable a portion of nerve, the continuity could have been again restored. Indeed, this is matter of demonstration, not merely of conjecture; for, in another part of the excellent work alluded to, an experiment is detailed, which sets the point at rest. A portion of one of the largest nerves in the axillary plexus of a rabbit was destroyed: the powers of the limb were gradually restored, and, at the end of seven weeks, no perceptible lameness remained. On examination, the extremities of the nerve were found totally unconnected for the space of half an inch. It remains, then, to be inquired by what means nervous influence is capable of being recovered, besides the restoration of interrupted continuity? Two ways present themselves in which this may be supposed to take place: one, that the nervous power travels to the lower portion of the divided nerve by anastomosing branches, as the blood does after the operation for aneurism; the other, that the branches of the nerves which remain perfect take upon themselves new vigour and new functions. Perhaps there is not yet sufficient data to determine between these two; but that it is not by a process similar to anastomoses, is rendered probable by the following experiment.* Two ligatures were applied to the sciatic nerve, which was then divided between them. About two months after, so considerable an improvement had taken place in the voluntary powers of the limb, as to render the experimentalist apprehensive that his endeavours to prevent reunion had failed; but in this he was deceived. The nerve was exposed two inches below the point of division; a ligature was applied to the most inferior part; "*but the animal did not evince the slightest evidence of sensation.*" It was cut with the same result; while lively sensation was manifested if the superior portion was similarly treated. Besides, that one set of nerves is capable, under certain circumstances, of assuming the actions of another, seems probable both from the result of experiment and the natural phenomena of some diseases. Dr. HAIGHTON has shown that, on the division of some of the nerves of voice, the others acquire increased energy, indepen-

* See PRING's *View of the Relations of the Nervous System in Health and Disease*, p. 36, 37.

dent of the re-union of the divided branches; and, in tic douloureux, the diseased action has been known to shift its seat instantaneously, in the manner of metastasis. Not only has the tic douloureux been known to change from one side of the face to another, but Chaussier has observed this metastasis between the infra-orbitory nerve and the foot.

If the nerves were all similar in their functions, this phenomenon would present an insuperable obstacle to the permanence of the relief afforded by division of the nerve for tic douloureux, even if its re-union could be certainly prevented ; but, if some nerves are destined for the powers of motion and others of sensation, it would hold out the greatest encouragement for the operation: for instance, if the fifth pair give sensation to the face, and the portia dura the power of motion, then a division of the infra-orbitary branch should be attended with no other change than substituting insensibility for acute suffering. But, as this is a branch of the subject which we mean, on some other occasion, to discuss at length, we decline entering upon it here. Indeed, we have already greatly exceeded the usual length of preliminary remarks, and must hasten to our analysis of the works before us.

The pamphlet of Dr. YEATS consists of a very minute and circumstantial detail of a case of neuralgia affecting the right thigh, leg, and foot; followed by some general observations. Mrs. Y— was attacked on the 7th of March, and took a variety of remedies without benefit, till the 3d of April, when she began the use of carbonate of iron ; and the report at half-past eleven next night states, that she was then freer from pain than on any day since her illness. From the 17th, her complaints gradually diminished; and by the 7th of June she was so well as to discontinue the medicine altogether. Although we have thus condensed the history of this case, it extends in the original through more than twenty pages, with a minuteness probably arising from the interest naturally taken by Dr. Yeats in the sufferings of his patient, who merits, we nothing doubt, the encomiums he has bestowed upon her patience and fortitude.

Before we proceed to the observations which follow, we must remark that sufficient pains has not been taken in correcting the press: at page 6 it is stated, that, "notwithstanding the great calmness of the pulse, it was impossible to divest one's self of the idea that there was *no* local inflammation in the coat of the nerve:"—*no* ought obviously to be *some*; and we cannot but regard so entire a perversion of the author's meaning as proceeding from a typographical error.

Dr. Yeats dwells at some length upon the variety of causes which may give rise to tic douloureux, and influence of the digestive organs upon the system at large, through the medium of the nerves; positions which no one, probably, will feel disposed to deny. He next adverts to the possibility of the state of the brain producing painful conditions of the sentient extremities of the nerves; but immediately adds, that this state of morbid condition of the brain may itself be only a secondary effect, of which the original cause is to be sought in the digestive organs. That it may be so with regard to many nervous affections, we do not deny; but, with regard to tic douloureux, many people labouring under it eat, drink, and digest, with a degree of industry and success that preclude the possibility of any defect existing in the system of digestion.

Several interesting cases are given in illustration of his opinions, and in particular of the necessity of attending to the exciting causes. "Having thus," says he, "ascertained, as well as we are able, and as well as the intricacy of the subject will admit, the source of the irritation, the *fons et origo mali*, the remedies at hand will readily supply us with means adapted to this consideration of the subject; and, if the complaint has not been one of long continuance, we shall, in many cases, be able to produce a fortunate result: but it will be necessary to ascertain the precise morbid condition of the organ which may be the original source of irritation; and this is, unquestionably, a task of considerable difficulty." Now, we grant that it is desirable to ascertain, if possible, the exact nature of the morbid changes which produce disease; but to do this is frequently a task, not of "considerable difficulty," but of absolute impossibility; an obvious instance of which presents itself in the subject of which we are treating. Neither, we fear, although we may succeed in discovering the precise morbid condition of the organ affected, will we always find the remedies at hand; for it is too true that the improvements in the practice of physic keep no pace with the advances made in pathological discovery.—We beg not to be misunderstood: we do not say this as undervaluing morbid anatomy; but we assert that there are yet many diseases, our treatment of which is purely empirical, and that it is better it should continue so, than be founded on speculative ideas of their nature, unsupported by demonstrative evidence, because we are more likely by many trials to find out the cure, than by much reasoning to find out the pathology.*

* We have been induced to postpone the review we have drawn up of the works of Mr. Hutchinson and Mr. Swan, finding that it would have occupied so much space as to exclude the foreign department altogether.

### DIVISION II.

### FOREIGN.

Art. IV.—*Memoires sur la Fievre Jaune considerée, dans sa Nature et dans ses Rapports avec ces Gouvernments.* Par N. V. A. Gerardin (de Nancy). pp. 91. Paris, 1820.

Art. V.—*Considerations sur la Fievre Jaune.* Par le Baron Larrey, &c. &c. Seconde Edition. pp. 42. Paris, chez Compeu, jeune. 1822.

Art. VI.—*Rapport presenté à son Excellence le Ministre Secretaire d'Etat au Department de l'Interieur, par la Commission Medicale envoyée à Barcelone.*—(Journal Génèrale de Médicine, Mars 1822.)

Art. VII.—*Manifeste touchant l'Origine et la Propagation de la Maladie qui a regné à Barcelone, en l'année* 1821; *presenté à l'auguste Congrès Nationale, par une reunion Libre de Médecins étrangers et nationaux.* Traduit de l'Espagnol, par J. A. Rochoux. pp. 35. Paris, chez Bechet, jeune. 1822.

We have been induced, thus early in our career, to undertake the consideration of the subject of Yellow Fever, partly from a conviction of its great importance at this moment,* and partly, also, in consequence of a promise which our predecessor has held out in a recent Number of this Journal, and which promise we were most anxious to fulfil. We approach the discussion with a deep sense of its difficulties, and, we hope, unbiassed by any particular theory. Recent circumstances have rendered it of such intense interest, and conflicting opinions have so obscured it, that, although we may hope to be pardoned if we fail to produce order out of this chaos, we should certainly have deserved reprehension if we had shrunk from making the attempt.

Some questions, alike interesting to the statesman and the philosopher, are involved in the inquiry concerning yellow fever; and upon the decision of one of these questions, at least, the propriety or necessity of imposing severe and irksome restraints upon a numerous class of the community entirely depends. This circumstance, which greatly enhances the value of the discussion, at the same time increases the difficulty; since the evidence that relates to this particular point is by far the most contradictory that offers itself to our notice.

The points to be resolved appear to be principally the three following:—1st. Is the disease that has of late years devastated Cadiz, Malaga, &c., and more recently committed such frightful ravages at Barcelona, the true yellow fever, or not? 2dly. Is it an imported malady? And 3dly, (which appears to be in

* The public prints inform us that this disease has again made its appearance in Barcelona.

some measure, but not entirely, dependent upon the decision of the former question,) Is it contagious, in the common acceptation of that word?—for the disease *might* have been contagious independently of any importation: neither does it appear to us that this latter circumstance, if proved, would be decisive of its character.

Before we introduce the works that stand at the head of this article to the notice of our readers, it may not be amiss to give a rapid sketch of the history and symptoms of the yellow fever of the West Indies and America, a subject illustrated by the labours of so many celebrated men, both English and foreign; and it is not a little mortifying to the pride of human learning to observe how few facts have been established, beyond the reach of contradiction, by the exertions of such an host of writers: yet, when we reflect upon the prejudices of education and of country,—when we consider how many enter into the inquiry with opinions already formed, and with the mental eye closed to every circumstance that tends to weaken their pre-conceived notions,—our wonder ceases, and we can only repose in the humble hope that *we*, who are so sensible of *their* errors, may happily avoid falling into the same mistake.

There can scarcely be found a more apposite illustration of the above remark, than the great variety of names that have been applied to denote this disease,—some imposed upon it in order to distinguish its supposed source or origin, as *the malady of Siam*, *the Bulam fever*, &c.; others from a leading symptom, as *the black vomit*, or *vomito prieto* of the Spaniards; others, again, from its supposed seat, as *la fievre gastro-adynamique* of Pinel; or, lastly, to suit some nosological arrangement, as Sauvages, who designates it *typhus icterodes*.

In tracing the history of yellow fever, it is curious to observe how very conspicuous a place the doctrine of importation will be found to occupy; and that attempts have been made, as early as the year 1690, to fix the origin of the disease upon the East Indies; but the argument in this instance is so well known to be contradicted by established facts, and the prior existence of yellow fever in the Brazils, at Martinique, St. Domingo, &c. is so amply proved, that it will not be necessary to recur to any authority to establish this point: indeed, two of the authors* who tell the story of the importation by the *Oriflamme* in 1690, give a different version of it; and, as there exists an accurate description of the fever that desolated the Brazils some years prior to this supposed event, and which description can leave no doubt as to the disease having been really the yellow fever, we may be excused from any farther research relative

* M. Bally and M. Moreau de St. Mery.

to this particular point. Since the date of the above story, a formidable list of authors, upwards of an hundred in number, may be found, who have successively laboured in this field, independently of the numberless essays and papers that have from time to time been inserted in the Transactions of the various learned Societies in Europe and America; some of them describing the disease generally; others, and by far the greater number, deriving their information, and giving their description, from some particular spot, or relating to the epidemic of a particular season; to which circumstance may be attributed the discrepancies that are to be found in the several accounts of the symptoms and progress of the disease.

The following is a brief sketch of the usual mode in which yellow fever makes its attack. Its first accession is denoted by cold chills or rigors, soon succeeded by intense heat and dryness of the surface of the body;* the face is red and flushed; the eyes have a peculiar and fiery expression, which has been compared to those of a man in a state of intoxication; violent pains are felt in the forehead and orbits, sometimes more particularly in the back and lumbar region; the countenance sometimes exhibits a remarkable expression of alarm; the tongue, at first moist, soon becomes loaded; the patient complains of nausea and tenderness in the epigastrium; troublesome eructations and vomitings of bilious matter quickly succeed, which, as the disease advances, become of a darker colour; the thirst is extremely great; restlessness and watchfulness distress the sufferer to a great degree; and this stage of the disease often lasts as long as two, or even three, days. The condition of the bowels differs very much: in some instances, constipation exists to a very remarkable extent. One of the distinctive marks of the complaint, mentioned by Mr. BALLY, is the length of time that the energy of the muscular power is sustained, so that a person shall be able to walk the street, or shave himself, within an hour of his death. At the termination of this stage, the more prominent symptoms, in general, remit: the patient and his friends are induced to believe that he has overcome the malady; but the listless and often torpid state of the patient, and a faint yellowish appearance about the chin, or on the sclerotic coat of the eye, too surely point out the danger that is lurking beneath this apparent calm. Dr. BANCROFT observes, as an alarming symptom in this stage of the complaint, that pressure made upon the region of the stomach will occasionally produce efforts to vomit; although the pulse shall have diminished in fre-

* HUMBOLDT tells a story of a traveller, who had passed a very short time at Vera Cruz, and, on his arrival at Xalapa, was told by his Indian barber that he would have the black vomit that evening; giving as a reason that the soap dried upon his face as fast as he applied it.

quency, the thirst and febrile heat shall have subsided, and even the intellects, if previously disturbed, shall have become clear. This, which may be called the second period of the disease, seldom lasts above two days, and is succeeded by renewed vomitings: the matter thrown up is streaked, or altogether black; passive hemorrhages from the mouth, anus, &c. supervene; the teeth and gums are covered with a black crust; the dejections become bloody, of a most offensive kind, and often involuntary; the urine is dark coloured, fœtid, and in very small quantity; petechiæ occasionally appear over the whole body, some hours previous to death. Swellings of the parotids, and of the axillary glands, are mentioned by some authors, but they do not seem to be essential to the disease: they were, however, met with frequently at Martinique, in 1802 and 1803.* The state of the intellects is by no means uniform: sometimes coma prevails; in other epidemic seasons, furious delirium has been more prevalent. The whole duration of the malady is from five to seven days, although many instances occur where death ensues within thirty-six or even twenty-four hours. The state of the pulse is represented as very variable. Dr. GORDON says that, at the commencement of the re-action, it is full and strong, but seldom exceeding ninety strokes in the minute. Dr. BANCROFT represents it as quick, though sometimes oppressed and irregular. At the end of the first twenty-four hours it increases in frequency.

It appears, by the concurrent testimony of some of the best writers, that yellow fever attacks the system most commonly between midnight and noon.

So much do these epidemics vary in their leading symptoms, that, in 1814, it is said that the black vomit was a rare occurrence. At Philadelphia, in 1798, the delirium was generally of a violent character. In some instances, a miliary eruption has made its appearance in the latter stage of the malady; and even the yellow suffusion is not always met with.† Examination of the dead body presents more points of difference than would at first sight be expected; and authors by no means agree in their accounts of the diseased appearances. These disagreements may, perhaps, be ascribed to the greater or less degree of severity of individual cases, or to variations in the epidemic constitution (to use an antiquated phrase,) of some particular seasons. Thus, whilst Bancroft declares that the brain has appeared to him more voluminous than natural, Mr. Bally has found it compressed by a red and bloody-looking serum; and Savaresi says that it is, in general, reduced to five-sixths of its usual volume. In some seasons, the lungs have been found

---

* M. de Jonnes.

affected, and the pleura inflamed; but the abdomen is the principal seat of the morbid changes, though even here we find a great contrariety of sentiment. Dr. GORDON has found the biliary organs frequently in a state of lesion; others have observed that the liver and gall-bladder remain in a healthy state, even where the stomach is loaded with the matter of black vomit. GERARDIN* has often seen the hepatic system unaltered; whereas ROCHOUX protests that there is no example of the gall-bladder remaining in a healthy state. It is admitted that the spleen and kidneys are generally sound; yet SAVARESI observed, at Martinique, in 1803 and 1804, that they were constantly affected. The mucous surface of the stomach and small intestines bears, however, the most unequivocal and universal marks of lesion, according to the unanimous testimony of all the best writers. Red and gangrenous spots are found scattered over their whole surface; and, finally, Dr. AUDOUARD informs us that, in numerous instances, the spinal canal contains a quantity of serous fluid.

We will not fatigue or insult our readers by quoting authorities to prove that yellow fever is indigenous in the New World; that it is of local origin, and can be fairly traced to the extrication of marsh effluvia, reigning sporadically, in a greater or less degree, among Europeans and new settlers; whilst the natives and the black population, excepting in particular seasons, escape with impunity, or, at most, only suffer partially and occasionally from slight remittent or intermittent fever. We are still, however, in darkness with respect to the causes which sometimes give vigour and activity to this poison at one period more than another, and which, after a few years' quiescence, render these climates so formidable to the inhabitants of our quarter of the globe; particularly hot seasons,—the fall of an unusual quantity of rain,—the direction of the winds,—the absence or presence of hurricanes, and other atmospheric phenomena, would probably, if duly registered and known, solve the difficulty. But this is a subject standing in need of much illustration, and the study of which we strongly recommend to those whose destiny carries them to these climates.

A careful inquiry into the topography of the different islands and places where the disease is to be met with, is also a great desideratum; although, since the year 1793, many important facts relative to this point have been noticed, both by English and foreign writers.† It is a pursuit of the highest importance, because it leads at once to the only remedies that can prevent a recurrence of the dreadful scenes that have been too common both in America and its islands; and which remedies, it has

* *Memoires sur la Fievre Jaune.*

† Ferguson, Bancroft, Watts, Revere, Gerardin, &c.

been, we think, satisfactorily shown, consist in ventilation, drainage, and cultivation. That, from the year 1793, in particular, such frightful mortalities should have occurred in St. Domingo and other of the Antilles, is not a subject of astonishment, when we consider the thousands of victims, in the fittest state to receive the disease, which the course of a sanguinary war poured out to these colonies.

We may now fairly proceed to examine the authorities on the much-disputed subject of contagion, which we shall do as briefly as possible, and then turn our attention to the disease which has appeared so frequently, of late years, on the coast of Spain and Italy; which will conduct us to the question of importation, and to its application to the recent case of Barcelona, in particular.

Several opinions appear to have been prevalent relative to the contagious character of yellow fever, by which term we understand a direct communication with the sick, or with the clothes, bedding, &c. of persons labouring under the disease. One of these opinions is, that it is contagious; another, that it is not; and a third and respectable portion, both as to reputation and numbers, hold a middle course, and believe that it is sometimes contagious and sometimes not so; whilst others, refining still farther, believe that, though not originally capable of communication, it may, under certain circumstances, become so, or that the contagious property has but an extremely limited operation in point of time as well as space. Among the contagionists are to be found the names of Lind, Lining, M'Kettrick, Batty, Chisholm, Pallone, Arejula, Pym, &c. The non-contagionists produce the names of Jackson, Moseley, Bancroft, Watts, Miller, Revere, M'Lean, Valentin, Savaresi, Deveze, Caldwell, Ferguson, &c. The partizans of the mixed opinion number among them Humboldt, Desgenettes, M. de St. Mery, M. de Jonnes (not a medical man), Clark: Baron Larrey and Gerardin must be also classed in this list.

With respect to Mr. Rochoux, we know not what to say; he seems to have changed his opinion so often, that it may fairly be doubted whether he has made up his mind as to which side of the question he finally intends to espouse. It is more than suspected that Dr. Rush, although he had formally abjured his belief in contagion, retained a strong predilection for that doctrine to his last hour.

In the very outset of the argument, it will be perceived that the non-contagionists have a manifest advantage over their antagonists, because one well-authenticated fact of non-contagion is, from its nature, of more value, than scores of cases of contagion as usually adduced; for, as these latter necessarily take place upon the spot which is the alleged source and origin of the noxious effluvia,—the very cradle of the disease, twenty men may successively fall ill from breathing the same atmosphere, without its being at all necessary to suppose they derived it from each other: nor does the exemption of secluded houses and families, even if the facts are admitted in their fullest extent, entirely clear up the difficulty; since it is well known that, in other cases of marsh fever, and upon sundry other occasions, the slightest difference of situation,—the interposition of a wall or dyke,—has been quite sufficient to preserve the atmosphere from contamination. In Walcheren, this was exemplified in numerous instances, especially at Fort Batz, where the troops suffered little or no sickness; whereas, those stationed without the fort, though only at a very short distance, were affected by the fever to a most alarming extent and degree. But what shall we say to the instance of New-York, in 1805, where a population of more than 10,000 persons, dreading the effects of contagion, fled from the town, and encamping at Greenwich, an elevated field at one extremity of the town, established their stores, banking houses, &c. on that spot, and where, finally, the customs and the courts of justice were transferred,—notwithstanding the constant intercourse between Greenwich and New York,—notwithstanding the importation of goods of all sorts, and touched and handled by all classes of people,—the disease did not spread in any one instance. Equally strong is the case of Leghorn, in 1804, when those who fled to Pisa did not communicate the fever; and, although two of those who removed there actually died of unequivocal yellow fever, no farther sickness took place. The same thing is recorded to have happened at Gibraltar, in 1814, by Mr. AMIEL.*

Mr. VALENTIN† has adduced numerous authorities, all concurring to establish the non-contagious nature of the disease. Dr. DUPUY, who witnessed both the epidemics of New Orleans of 1819 and 1820, not only is of this opinion, but declares that all the practitioners, with the exception of two or three, agree in this point. In contradiction to Dr. Gerardin's implied meaning, he says, that those who fled from the city to other parts did not communicate the complaint to the inhabitants of those places to which they fled. The mortality of the epidemic of 1820, in particular, was so dreadful, that, of those attacked by it, seven out of ten died.

It appears, also, that Dr. CHERVIN, then at New Orleans, had, after witnessing the ravages of yellow fever at Guadaloupe, and actually examining more than four hundred dead bodies, made a tour of the Antilles, and of many parts of the United

* *London Medical and Physical Journal*, July 1815.

† *Journal Generale de Médecine*, Jan. 1822.

States of America, in order to collect the opinions of the profession upon the subject of contagion: the result is, that, out of about 150 certificates which he obtained, there were not above fifteen who adhered to that doctrine. Dr. Chervin is now at Paris, preparing his materials for publication again. A committee of seven physicians was appointed to examine into the causes of the epidemic at Mobile (Florida): their opinion as to its local origin is unanimous, and most satisfactory. Such also is the result of the researches of Dr. CHALARD, of Baltimore.

Several other analogous authorities are adduced by this able and zealous writer; but it is useless, we conceive, to accumulate farther evidence, which can only tend to swell this article and tire the patience of the reader. As a specimen, however, of the credulity of the contagionists, we may here mention a fact brought forward by Dr. PYM,* and which, we think, can only excite a smile. He states that a man of De Rolle's regiment, in leading a comrade affected with the fever, at Gibraltar, to the hospital, turned sick, and expired on the road; and this is adduced as a proof of contagion.† A much stronger circumstance is related by Baron LARREY; it is this: Dr. VALLI, a few days after his arrival at the Havannah, took off the shirt of a sailor who had died of yellow fever, rubbed his own body with it, then put it on, and went to dinner with his host, Don Gonsalez. He remained quite well the next day; but, on the day following, he was taken ill, and died in twenty-four hours. Now this *appears* quite convincing: yet, when we consider that Dr. Valli was just arrived from Europe, and that yellow fever existed at the time, much of the force of the above case is destroyed; and it remains at best but very equivocal evidence. GIRARDIN also tells us that yellow fever raged at Natchez, Batonrouge, and other places, at the time of the epidemic of New Orleans in 1820; although some of these places, the former in particular, is remarkable for the healthiness of its situation, and is distant 150 miles from the source of the malady: but he admits that all these places were crowded with those who fled from New Orleans; and he does not inform us whether the disease was confined to these refugees, or whether the untravelled and original inhabitants suffered by the arrival of the strangers. This is, indeed, evidently implied in the account, and is in consonance with his own belief and opinion, but he has left the matter in great doubt.

It would be injustice, in this place, wholly to pass over the strong facts and arguments adduced by Dr. FERGUSON, in corroboration of what has been stated above, but it will be sufficient

* PYM on *Bulam Fever*, p. 24.

† LARREY, *Cons. sur la Fievre Jaune*, (note.)

for our purpose to notice two or three of the most striking illustrations he has given us in support of the non-contagious nature of yellow fever, without entering into the merits of his opinions upon other points of the argument. The first of these facts is the exemption which all the inhabitants of Monk's-Hill Barracks, Antigua, enjoyed during the epidemic of 1816, whose duty did not oblige them to sleep out of that garrison; whereas the soldiers who mounted guard at the dock-yard, and in other low situations, were often seized, while on their posts, with the most aggravated form of the disease; many dying within thirty hours from the first attack. Another important observation goes to prove that a slight elevation in the immediate vicinity of a marsh is more fatal sometimes than the ground upon a level with it, the higher ground appearing to attract the effluvia. This is in conformity with our own experience in the case of intermittent fever in many situations in this country.

The neighbourhood of trees is also observed, by this author, to afford a protection from the poisonous effects of marsh effluvia; and he gives us the example of New Amsterdam, where fever does not prevail, although it is situated within a stone's throw of a most unwholesome swamp, with a strong trade-wind blowing day and night from it towards the town, and without any other protection than this screen of trees: yet it is found that sleeping under them, or remaining there after sun-set, is almost certain death to any European. It may be added, that the inhabitants are well aware that their exemption from fever is owing solely to this cause.

Upon the whole, then, the conviction upon our minds arising from all we have read and thought upon this subject, is, that yellow fever is not a contagious disease; that it is of local origin; that it exerts its energy principally during night, at which time a very transient and temporary exposure to its causes is sufficient to light up the flame in a habit predisposed to receive it; and that although, from causes yet unknown, it acquires such fatal activity at some particular seasons, it is always to be met with as a sporadic affection in those climates, and, if we may believe some authors, even at our own doors.* Notwithstanding all these articles of faith, we are, however, willing to admit that the conviction entertained by some very judicious practitioners, that, although not originally or necessarily contagious, yellow fever may, and does, occasionally become so, is not to be altogether despised; since there does not appear any thing unreasonable in the supposition, that crowded habitations, poverty of living, and personal uncleanliness, (to say nothing of moral causes,) may so concentrate and condense

* VALENTIN says that sporadic cases are met with at Brest occasionally.

the poisonous effluvia as to superadd a contagious property to a disease originally free from it. We do not *know* this to be the case; but some circumstances within our own recollection, occurring to certain portions of the peninsular army, as well as consequent upon the unfortunate expedition to Walcheren, give some colour to this argument, and lead us to suspect the existence of what an able contemporary has denominated *contingent* contagion.* The subject is confessedly one of great difficulty; but it is not a mere question of the schools, since upon the belief of contagious diseases depends the propriety of imprisoning a whole population; and we cannot but think it to be abundantly proved, that both humanity and policy are equally outraged by the adoption of measures of such extreme and useless rigour. If, as in the case of New-York, the *healthy* population were removed out of the sphere of the malady, which *we know* to be of local origin, we conceive that a stop would at once be put to the spreading of the evil; whereas the removal of the *sick* tends merely to increase the alarm, and leaves only a succession of victims to be swept off, as long as the influence of the miasmata remains in activity. Still more cruel is the close circumvallation of the whole population, which, after all, cannot be so complete as to baffle the courage and ingenuity which the dread of so formidable a disease will frequently inspire.

Before we proceed to the direct question of importation, there are a few interesting facts which must be mentioned, and from which it would appear that a disease similar to yellow fever in all its leading symptoms, and unfortunately also in its fatality, has, upon certain occasions, been produced on-board ship, without the most remote possibility of supposing it to have been exported from any of the known sources of that malady. The most remarkable of these events is recorded by M. Beguerie,† who has seen the yellow fever in the West Indies, and experienced an attack of it in his own person. It appears from his account that a French flotilla, with troops on-board, which sailed from Tarentum for St. Domingo in 1802, after having been driven about the Mediterranean by stress of weather, and having been obliged successively to put into the ports of Leghorn and Carthagena, sailed from this latter place in the month of August. The heat of the weather had been dreadful in the months of May, June, and July; the provisions on-board are represented as of the worst quality, and the salt fish in such a state of putrefaction, and giving out so horrible a stench as to oblige them to have it thrown overboard. A fever broke out

* *Medico-Chirurgical Review*, March 1822.

† *Histoire de la Fièvre qui a regné sur une Flotille de la Montpellier*, 1816.

on-board this fleet soon after they sailed, and lasted until their disembarkation, acquiring force as they approached the tropic. M. Beguerie assures us that the symptoms of this fever differed only from those of yellow fever *by an almost imperceptible shade.*

In the month of August, 1802, an American vessel arrived at Marseilles from New Providence, after having touched at two Spanish ports. No epidemic reigned at either of these places; neither had she any sick on-board during the passage, nor during a quarantine of fourteen days; but, after the crew had disembarked, the second and third officers and four sailors were successively attacked with yellow fever, and died,—three in different houses in the town, and three at the lazaretto; but it did not spread either among the inhabitants or to the physicians and attendants of the sick. The writer of this article* is therefore induced to believe that, in this instance, the vessel must be compared to a spot where the air is hot, moist, and stagnant, and rendered impure from many sources of corruption.

One more very strong instance of the development of yellow fever on-board ship is recorded by Dr. FERGUSON:† it is the case of the *Regalia* transport, which was employed in bringing black recruits from the coast of Africa to the West Indies, in 1815. This vessel is represented as leaky, and having taken on-board a quantity of green wood in Africa; her ballast also was foul, and had not been changed from her quitting England, nor for any discoverable time previously. The black recruits were crowded in this vessel, many of them afflicted with fluxes, ulcers, &c. The provisions were defective both as to quantity and quality; and the crew, prior to their sailing from Africa, were healthy. It appears authenticated that this ship arrived at Barbadoes with yellow fever on-board, in the month of August; that, owing to some negligence, she was not put under quarantine, but communicated freely with the Saints, Antigua, and Guadaloupe, landing those dying of the disease among the inhabitants and at the hospitals of those places, without communicating the disease at either of them; and, finally, after having undergone a thorough purification, sailing from Guadaloupe to Europe, crowded with French prisoners and their families from the jails, under the most dangerous circumstances of health, with a case of yellow fever dying on-board the day before she left Basse-terre Roads; but without any contagion spreading to the other passengers, and without importing it at the port which she ultimately reached.

Dr. LEFORT,‡ of Martinique, in a letter to M. Valentin, states

* M. Fournier.

† *Medico-Chirurgical Transactions,* vol. viii. p. 109.

‡ *Journale Generale de Médecine,* Jan. 1822.

that the yellow fever broke out spontaneously on-board a vessel called the *Euryalus*, cruising in the tropical seas, without having touched at any port in those seas; and this he declares to be the fifth instance of the kind, within his own knowledge, in a period of four years.

M. Moreau de St. Mery adduces the following fact as an unanswerable argument in favour of contagion: we do not see it in that light; however, we are bound in candour to relate it. The *Palenicrus*, a French brig, having the yellow fever on-board, and cruising in the West Indies, encountered the English brig *Carnation*, coming direct from Europe, the crew quite healthy. A combat ensued, and the English brig was captured (a rare occurrence,) by boarding: the English sailors were consequently removed as prisoners into the French ship, and took the disease. Now, we think this and other examples quoted above will enable us to clear up this difficulty, without the necessity of recurring to contagion; for, if the French vessel was the focus of the yellow fever in this last instance, she would stand, in relation to the English sailors, exactly as a village or town surrounded by a contaminated atmosphere would stand with respect to its inhabitants, or to strangers arriving there from other quarters: but if, on the contrary, a French sailor labouring under the yellow fever had been sent on-board the English ship, and the disease had spread amongst the healthy crew, we should then be under the necessity of admitting that a case of contagion had been made out, beyond the reach of cavil or dispute.

A late Number of a foreign periodical work,* contains an account of a sickness occurring on-board a vessel called the *Arthur*, which sailed from Rouen in 1818, laden with *poudrette*, a species of compost made from human ordure. On the voyage to the West Indies, a disease broke out among the crew, of so alarming a nature, that one-half died on the passage, and the rest arrived at their destination in a miserable state of health. Those who unloaded the vessel suffered equally from the same disease. M. Parent, who was deputed by the French government to trace the cause of this accident, discovered that a similar fate had attended the crew of a little bark laden with the same material at Nantes; although the workmen, who prepare the article upon a very large scale, and who perform the process in the *open air*, are found to be remarkably healthy. The nature of the disease produced in both these instances was a fever of that type called by the French *fievre adynamique*, the most prominent symptoms being head-ach, pains in the limbs, fever, nausea, and vomiting. It is not stated whether any of the crew of the last-named vessel died; nor do either of these instances

* *Bibliothèque Medicale*, Mai 1822.

go the whole length of proving that the malady produced was actually the yellow fever; but they establish this fact, that the putrefaction of animal and vegetable matter, aided by warmth and moisture, is capable of producing a disease resembling yellow fever in some of its most prominent features, as well as in its ratio of mortality.

From a due consideration of the foregoing facts, and many others equally strong, resting on authorities the most respectable and undoubted, we do not perceive any thing paradoxical in the assertion, that, whilst we believe yellow fever to be in its nature non-contagious, we are clearly of opinion that it may be, and often has been, imported. That it has ever spread by importation, or that the mortalities that have occurred so frequently in the New World, as well as in Europe, are to be ascribed to this source, we most positively deny; but when, from a concurrence of local causes, an epidemic has broken out, and it becomes an object to trace it to some palpable and known origin, can we be surprised that it should be discovered to have existed on-board some ship from the West Indies, or that it should have developed itself on-board some vessel during her passage? In fact, numerous examples of the sort are upon record; but does this circumstance establish a necessary connexion between the disease on-board and the epidemic on-shore? We think decidedly not; for repeated experience has shown that men brought from on ship-board, and dying of yellow fever at different houses, have not spread the disease to any single individual; and, again, epidemics have sometimes raged, of the importation of which not only no evidence is offered, but not even any suspicion existed. The shores of the Mediterranean have enjoyed an exemption from this calamity for many successive years. Is it reasonable to suppose that, in all that time, the quarantine laws have never been violated or evaded, when such instances are discovered to be of every-day occurrence, when they are wanted to be brought forward as evidence of the foreign origin of the complaint? But, in truth, such inquiries have never been thought of until the breaking-out of the fever has given rise to them; and, when instituted, if a solitary case of yellow fever has been traced to have occurred within a short period of the invasion of the malady, the problem has been considered as solved, and all collateral and minor evidence tortured to meet this explanation. But, if we believe that, in any one well-attested instance, yellow fever has been traced from a West Indian or American vessel without communicating contagion, the argument in favour of that doctrine is at an end, and importation may still be credited without considering contagion as a necessary consequence.

The course of our narrative has now brought us to the consideration of those frightful scenes which have occasionally been exhibited on the shores of Italy and Spain, but which have, since the year 1800, been not only of more frequent occurrence, but more fatal in their results. That Cadiz, Carthagena, and Malaga, have been visited three or four times in the course of the eighteenth century by destructive epidemics, and that these epidemics were really the yellow fever, there can be no manner of doubt. The writings of contemporary authors are conclusive upon this point, and Lind was himself a witness to one of these visitations. The more recent occurrences at Cadiz and Gibraltar have given birth to such numerous testimonies of the most respectable kind, that there can be no hesitation in asserting that the first of our questions is satisfactorily answered in the affirmative; and nothing now remains for us but to examine the documents that relate to the fever at Barcelona in 1821, and the only point of accordance that can be discovered between the various individuals who have so zealously devoted themselves to the contemplation of this malady, is the undoubted fact of its having been the yellow fever.

We shall now, without farther comment, proceed—1st, to lay before our readers, as succinctly as we are able, the substance of the Report made by the French commissioners to their government relative to this epidemic, as bearing the stamp of authority; and afterwards present them with a Manifesto, published by the spontaneous union of several physicians, both English, French, and Spanish, in Barcelona, and which is, in fact, a direct contradiction to all the assertions contained in that report, without having been originally intended as such; since, at the time of its publication, the report of the French commissioners was not known at Barcelona. In the course of this analysis, all the facts connected with this melancholy visitation of Providence will become developed; and the conclusions which we think must inevitably result, will tend, in a very satisfactory manner, to confirm the opinions attempted to be maintained in the former part of this paper.

With regard to the report of the French commission, it is but cold language to say that it is one of the most extraordinary documents ever presented to public notice: it would, perhaps, not be too harsh to affirm that it is also the feeblest in reasoning, —the weakest in fact, and the strongest in assertion, that ever issued from the press. It bears the most decided marks of preconceived opinions, but is, fortunately, so hastily and crudely put together as to carry the conviction of its weakness in every page: in short, it displays a determination to discover, what we are persuaded it was intended to find, an excuse for a sanitary cordon. The gentlemen composing this commission were originally five in number,—namely, Messrs. Bally, François,

Pariset, Mazet, and Rochoux; the whole of them, with the exception of the last, most decided contagionists,—a circumstance which alone will afford a tolerable guess at the impartiality that may be expected from an inquiry conducted by such a junta. Their personal narrative is shortly thus: They quitted Paris on the 28th of September, and arrived at Barcelona on the 9th of October, at seven o'clock in the evening; and, by half-past eight, they had begun their labours, and visited some sick.

As the commission was so soon to be freed from the presence of M. Rochoux, we shall dismiss him at once, giving the motives of his secession as represented to us by his companions, and which, if correct, is ludicrous enough. He escaped by a piece of logic. "The fever that rages at Barcelona," he said, (we quote the words of the remaining commissioners,) "is either the yellow fever of the Antilles, or it is not: if it is, it has no contagious property, as we shall see; but, if the disease has any thing contagious in its nature, I am not sent here to study a malady of that kind, and therefore I shall separate myself from you immediately." In consequence of this opinion, they assert that M. Rochoux retired to Garcia on the 14th, and, after divers projects, separated himself entirely from his comrades. They more than insinuate, that M. Rochoux was induced to adopt this line of conduct in consequence of the death of M. Mazet, which took place on the 22d instant, after an illness of nine days: he was taken ill in the night between the 12th and 13th, having only seen and touched two sick persons. It is, however, but justice to M. Rochoux to observe, that, as the commissioners have been detected in perverting the truth with respect to another of their countrymen,* (who, indeed, has proved the fact against them in the most unanswerable manner,) we are therefore bound to give that gentleman the benefit of the doubts which such unfair and illiberal conduct has necessarily excited in our mind.

The commissioners then continue their narrative as follows: —Dr. Audouard, who was sent to Barcelona by the minister of war, arrived there the day after M. Mazet's death; but he did not join them, he established himself at the botanic garden. They declare that they met him but seldom; that he was accustomed to work independently of them; which, joined to the sickness of two of their number, separated them from each other, without, however, causing any division between them. Their history then concludes with some account of the mode of conducting their researches. In the night of the 24th, Messrs. Bally and Pariset were attacked with the disease; M. Bally

* Dr. Audouard.

suffered most. During their secession, M. François continued his visits, and made the first *examinations of dead bodies;* for, until then, they had no instruments. An assistant fortunately came from Perpignan, a M. Jouarii, "poor, but full of zeal." Morning and evening he attended the visits of M. François, and in the day wrote what was dictated to him by M. Bally. This latter gentleman, when enabled to go about, employed him especially in anatomical examinations. From the 6th to the 19th of November, M. Bally resumed his duties at the hospital, and between these dates the clinical observations and dissections are represented as having been more regular and complete. On the 20th of November they finally quitted the place, their health beginning to suffer again.

We must stop here one moment for the purpose of reviewing the last paragraph; and it will hardly be believed, yet such is the fact, that Dr. Audouard* declares that M. François never put his hands into the dead bodies at all; that, instead of seeing each other but seldom, he met them every day; that the first dissection made in the Hospital of the Seminary was made by him, on the 31st of October; and that M. Bally did not open any bodies until the 8th of November; whilst Drs. Revera and Campmany, whose names the commissioners do not even deign to mention, had pursued their anatomical investigations throughout the month of August.

Having now sketched the personal adventures of these gentlemen, we come to the substance of their researches: they begin by declaring the salubrity of the situation of Barcelona, but especially of Barcelonetta, the streets of which town are wide and regular, and the foundation a bed of granite rock. They, however, confess that remittent fever occasionally reigns at this place. With regard to the condition of the port, they assert it to be perfectly clean, and that the water is pure, clear, and limpid: there are certain pools of stagnant water upon the beach, to be sure, but then they are only a few toises in extent, and but three inches deep! They next call to their aid the evidence of M. Simiane, captain of the French brig *Josephine,* who is destined to make a considerable figure in this history. This connoisseur in stinks is introduced for the purpose of proving that the morning wind, which regularly blows from the town, and which constantly conveys all the emanations from the city to the shipping in the harbour, never brought with it any odour which displeased *him.* Now, without disputing this gentleman's taste, we cannot help thinking that the odour from the cleanest city in the world cannot be fragrant; but, when that city is a Spanish one, we can only pity the cause which is

* *Lettere à Messrs. Pariset, &c.*

obliged to have recourse to such feeble support. It appears that the streets of Barcelona are narrow and tortuous; that they are traversed by canals, which receive the filth of the city to convey it to the sea: these canals are covered with large stones, but so badly joined, that any odour may readily escape and mix with the air. These inconveniences, they allow, are sufficiently unpleasant where the temperature of the air is so high, but they are not much felt excepting after rain, and in Catalonia this does not often occur. They admit that it did rain for some days after their arrival; but they forget to mention the fact of the great increase of sickness that immediately followed those days of rain, and from which the ignorant had expected great benefit. However, they get rid of these suspicious circumstances by reminding us that Barcelonetta was visited by the fever before Barcelona. The thermometer, which had been, during the months of April, May, and June, and part of July, never above 15° of Reaumur, rose at this latter period as high as 22°. On the 12th of July, the fête of the Promulgation of the Constitution was to have been held, but, as the weather was bad, it was put off until the 15th. The weather being remarkably fine, on that day the whole population was poured upon the ramparts, the quays, and the vast esplanade of Barcelonetta; the vessels in the port were also crowded with spectators. At this period there were a great number of ships in the harbour, both Spanish and others, recently arrived from the Havannah and Vera Cruz. Some of these had suffered from yellow fever at those places, some on the passage; the dead had been thrown overboard, but their goods, clothes, bedding, &c., covered with black vomit, had been preserved on-board. The eternal M. Simiane is again brought forward to prove that these things were exposed to the open air under his own eyes; although the captains had the art to elude the vigilance of the medical police, and contrived to attribute all the deaths that had occurred to falls from the masts, or other accidents. In order to avoid the quarantine laws, the sick were forced to shave and dress themselves, and appear on deck among the crew and passengers, as if in perfect health; which proves, at least, that neither the crews nor the passengers had found much reason to dread the contagion.—Now the marvellous part of the story is, that M. Simiane saw all these things, which the medical police either could not or would not see; and yet this vigilant and all-seeing captain never said one word upon the subject to any but to these commissioners, and that in the month of October, although the Havannah fleet arrived at Barcelona thirty-three days before any sickness was even *talked of* at that place.

To continue.—On the 15th of July, all the vessels in the harbour were crowded with spectators, and it may be *supposed*

(mark this word again in an official paper!) that many of the women and people passed the night on-board, stretched upon the mattresses and coverlids of those who had died. The first ship they mention is *Le Grand Turc*, which arrived at Barcelona on the 29th June, 1821, in sixty-one days from Havannah. A little while after, the captain, M. Sagreras, brought his family on-board, which family resided at Sitjes; they staid there but one or two nights: on quitting the vessel they were taken ill, and all, comprising the wife, children, and a female servant, died at Barcelonetta. Now this would, indeed, be an afflicting tragedy and a strong case, but for one trifling circumstance,—not one word of this story is true; and M. Sagreras, his wife, children, and maid, are, we feel pleasure in saying, all alive and well, and, what is still better, have not had the fever at all. This fact is stated upon the authority of M. Zaba, a merchant of Barcelona, an intimate friend of M. Sagreras. It seems that M. Rochoux was himself the author of this strange mistake, which he communicated to M. Pariset; and therefore, though the commissioners are to be acquitted of any intention to deceive, they have given us, by inserting this story without making proper inquiries as to its authenticity, an additional proof of the eagerness with which they seized upon every thing which could tend to confirm the opinions they had previously adopted. It is farther asserted, that thirty-five of the people who had been on-board this ship on the 15th of July, died a few days after. Now here again they make an unfortunate mistake; for no epidemic sickness occurred in Barcelona until the 3d of September, just fifty days after the day of the fête. This is proved by the official documents published by the municipality.

We must now proceed to dissect their second story, which relates to a vessel named the *Nuestra Senora del Carmen*, sixty-three days from the Havannah, and having touched at the ports of Alicant and Carthagena. She arrived at Barcelona on the 11th of July. Three of the crew had been ill with yellow fever at the Havannah, and one had died; the remaining three, it is added, had *probably* had the disease, as they had been to America before. Now observe what follows: This ship had received a poor passenger on-board at Alicant, for the purpose of conveying him gratis to Barcelona. Two days before the vessel reached that place, this poor man fell sick: and this is the person alluded to as having been obliged to dress himself, and appear upon deck as if in health. On the evening of their arrival he was disembarked, and died the next day. Had this man the black vomit? Ask the commissioners, and they answer their own question in the following satisfactory and philosophical manner. Many people, they say, affirm that he had; but, at all events, it cannot be denied but that so mortal a malady had a great affinity to yellow fever! They also think it not unreasonable to *suppose* that his disease must have propagated itself in the house where he lodged; for, being poor, with what could he repay the hospitality of those who received him?—With his clothes; and no doubt they were made use of by this family.

Let us now just recapitulate this precious, this unique piece of evidence. Nothing is known of this poor man beyond the fact of his having been brought from Alicant, and dying at Barcelona the day after his arrival; but, by the aid of three or four suppositions, he is convicted of having died of the yellow fever,—of having communicated the disease to the inmates of the house where he lodged, and that by means of his clothes with which they *suppose* he paid for the hospitality he received.

One more circumstance, and we have done, and this relates to our old friend M. Simiane, who, it is asserted, although in health himself, communicated the disease to the landlord of the house at Barcelonetta in which he lodged.

The succeeding pages contain some information relative to the number of deaths during the whole course of this epidemic, and they are calculated at from 17 to 18,000. During its greatest height, from 450 to 500 dead were carried out of the different gates of the city in one day. In this instance, as in all previous ones, it was observed that the bakers, and those whose occupations exposed them to great heat, suffered especially; whilst those who were addicted to excesses of any kind were most liable to be attacked. Contrary to what is usually observed, strangers from the northern countries of Europe especially did not appear so obnoxious to the influence of the fever as the natives.

We trust that we may be excused from pursuing the details of this report any farther, especially as we must now bestow a little attention upon the Manifesto we have already alluded to, and which will afford abundant and direct contradiction to those assertions of the French commissioners which want of time and space oblige us to pass over; but, before we remark upon that production, we beg to make an extract or two from the recantation of M. Puguillem, formerly a decided contagionist, but whose name is to be found attached to the manifesto of the adverse party, and whose abjuration is addressed to Dr. Lassis.* Among the motives which induced this gentleman to change his opinion are the following: "The regular march of the fever from east to south-west,—the existence of sick in different parts of the town before all communication with Barcelonetta was cut off,—its attacking those who were most rigidly sequestered,—the fact of several individuals in one house being seized with it

* *Diario di Brusi*, 22d December, 1822.

at the same time. Messrs. Bally, &c. (he continues,) went about seeking only those facts which appeared to favour their own cause; and, in reply to their remark, that one positive fact is equal to thousands of negatives, he says that their positive facts lose all their force when submitted to the test of rigid criticism; a point which, unless we deceive ourselves, we have sufficiently shown above.

In the Lazaretto, in the Hospital of the Seminary, in the General Hospital, neither the medical men nor those who attended the sick suffered the slightest attack. The sisters of the General Hospital escaped with perfect impunity; whereas the purveyor, the apothecary in chief, and others who never entered the wards, and who studiously avoided all contact with the sick, experienced an attack. It is impossible to find, he adds, one single well-attested instance of a sick person quitting the town, and spreading the disease to any of the neighbouring communes; and he urges the strong fact of the inhabitants of Sans, Garcia, and many other places, having escaped the disease, notwithstanding they were included within the cordon which enclosed Barcelona. Hence he thinks it extremely unlikely that contagion could be brought from the Havannah, when even the small distance of Garcia from the city was found sufficient to impede its communication.

The last remaining document (the Manifesto,) is signed by the following gentlemen: Dr. Maclean, Dr. Lassis, Dr. Rochoux, Francisco Piguillem, Francisco Salva, Manuel Duran, Juan Lopez, Salvador Campmany, Ignacio Porta, Jose Calveras, Antonio Mayner, Raymuno Duran, and Benaventura Sahue. Upon the authority of these names, it is asserted that sporadic cases of yellow fever were met with both at Barcelona and Barcelonetta, as early as February and March of the year 1821; and Dr. Lopez himself was called in consultation to a man who dwelt behind the Exchange, and who died of yellow fever, with petechiæ and black vomit, in the early part of February; and it will be recollected that the accused vessels did not reach the port until the latter end of June. After the disease broke out, numbers of sick retired to Sitjes, Malgrat, &c. but no sickness ensued at those places.

With regard to Tortosa, where the fever was supposed to have been introduced by a dealer in hams, the Junta of Health affirm that, many days prior, a sick man was brought from on-board a bark that had never been at Barcelona. It is to be remarked also, that towards the end of summer, that town is always visited by fevers of a very violent character.

With respect to the state of the port, it is asserted, that such was the condition of the sewers, the canals in the streets, &c. that, towards the end of June, it was impossible to pass along the sea-wall without being inconvenienced by the stench produced by the decay of animal and vegetable matter in its vicinity. The examination performed by the commission charged with cleansing the port, proves that the Arequia was obstructed at its mouth by a sandbank, which had caused the accumulation of a mass of stinking water, loaded with the impurities furnished by all the manufactories, slaughter-houses, &c. situated upon the banks of this rivulet, from whence a most insupportable stench arose. The modern works of the port appear to have increased the evil, and have produced a source of infection which did not formerly exist.

The mortality was most especially great in those streets in the line of the port; whereas, in those exposed to the north, and more distant from the infected spot, but few sick were found.

The time of the year in which the fever broke out is precisely the period in which epidemics make their invasion in hot countries: this fact has been verified more than once in Spain.

The fever has not been able to establish itself beyond the walls of Barcelona. No person has been proved to have caught the disease out of the sphere of the operation of local causes.

At the Marine Lazaretto, between the 7th of August and the 13th of September, 79 cases were received, 55 of whom died: not one individual, out of 32 employed in that establishment, took the disease. At the Seminary, 1767 sick were admitted, 1293 died; but only three cases of the fever occurred among 90 people employed in that establishment.*

M. Ribera, in dissecting a body, wounded his finger deeply with the scalpel: nothing beyond a slight swelling of the axillary glands took place.

Many, who had suffered from the disease in America, contracted it again, and some of these died.

Many families, who secluded themselves in the most rigorous manner, found their precautions in vain.

At the time the barrier was placed at Barcelonetta, on the 3d of September, there were only nine sick in the place: on the 10th instant, they amounted to 162; and, finally,

Those who had quitted the place with all their effects did not spread the disease at their new residences; although some few died of yellow fever, which they carried with them.

Such, among many others, are the strong, and we think unanswerable, facts which the perusal of this able paper has enabled us to lay before our readers, as confirming the view we have taken of this complicated and highly interesting subject. We, perhaps, have been tedious, and have accumulated evidence which many may consider as redundant; but we were anxious

* M. Jouarii made the fourth.

to collect in one view all the most important remarks which the experience of the last fatal year had produced; in doing which we have been as careful as possible to exclude all doubtful evidence, on whichsoever side of the question it seemed to bear. In the same spirit, we must therefore remark that, whilst we agree with the authors of the Manifesto in most of their views, we were sorry to notice some allusions to the plagues of London and Marseilles, which we think they have unnecessarily introduced in that paper: they savour rather too strongly of the doctrines of the gentleman whose name appears at the head of the list of signatures; doctrines to which we cannot subscribe, since we believe the plague to be a disease quite distinct in its nature, and we are not prepared in this instance to dispense with those precautionary measures, by means of which, we firmly believe, Europe is indebted for its long exemption from the visitation of that tremendous malady.

Having anticipated in the foregoing pages nearly all that is necessary to be said respecting the works at the head of this article, we have only to say that Dr. GIRARDIN deserves the praise of having given us, in his little memoir, a very accurate topographical account of Louisiana, and the neighbourhood of New Orleans in particular; an example which we hope will be followed by all those who undertake to discuss the subject of all epidemics, wherever they are to be met with. It has been already shown that the Doctor is a modified contagionist. In the latter part of his book he notices, and recommends to the ruling powers, the necessity of stationing raw European troops in such situations, in the different islands, as may ensure them from the operation of the local causes of the disease.

Baron LARREY's paper is principally remarkable for his speculative doctrines with respect to contagious virus, of which he distinguishes two kinds,—the fluid, and the gaseous or miasmatic: the former are the syphilitic, the small-pox, and vaccine poisons; in the latter class he ranks the yellow fever. The venereal virus, he goes on to say, chiefly affects the lymphatics; it can remain a long time inactive in the system, but, when it begins to act, it continues its progress unto the death of the patient, unless arrested by curative means. The variolous poison has a particular affinity to the skin: it is capable of producing a similar disease, but only for a determinate period, after which it becomes inert. The pestilential virus acts chiefly upon the brain and nervous system, though it is occasionally arrested at the nervous plexuses of the armpits and groins; and M. Larrey does not believe it to have any connexion with the lymphatic system. The virus of the yellow fever he considers as the most subtile and volatile of the whole; that it has but a momentary existence, and which corresponds to the acmé of the disease. The virus resides in the cutaneous transpiration, or in the eruptions when they are met with.

We see in this attempt of Baron Larrey's a love of generalization and system, to which we think our zealous and imaginative neighbours are too much addicted. It would be easy to contest this explanation step by step, but we have already devoted so much space to this discussion, and we think the weak points of this arrangement so evident, that it is scarcely necessary to point them out to the medical reader. The just reputation that M. Larrey enjoys rendered it incumbent upon us, however, to state his opinions; and we need hardly say that, in conformity with those opinions, he advocates the propriety of precautionary measures.

